# Consumption of red, white, and processed meat and odds of developing kidney damage and diabetic nephropathy (DN) in women: a case control study

**DOI:** 10.1038/s41598-024-59097-1

**Published:** 2024-05-06

**Authors:** Atieh Mirzababaei, Faezeh Abaj, Zahra Roumi, Reza Amiri Khosroshahi, Yasaman Aali, Cain C. T. Clark, Mina Radmehr, Khadijeh Mirzaei

**Affiliations:** 1 Department of Community Nutrition, School of Nutritional Sciences and Dietetics, Tehran University of Medical Sciences (TUMS), Tehran, Iran; 2 Department of Nutrition, Electronic Health and Statistics Surveillance Research Center, Science and Research Branch, Islamic Azad University, Tehran, Iran; 3 Department of Clinical Nutrition School of Nutritional Sciences and Dietetics, Tehran University of Medical Sciences, Tehran, Islamic Republic of Iran; 4 Centre for Intelligent Healthcare, Coventry University, Coventry, CV1 5FB UK; 5 Department of Nutrition, Science and Research Branch, Islamic Azad University, Tehran, Iran; 6 Department of nutrition, Dietetics and food, Monash University, Clayton, Australia

**Keywords:** Diabetic nephropathy, End-stage renal disease

## Abstract

Diabetic nephropathy (DN) is one of the most prevalent and severe complications of diabetes mellitus (DM) and is associated with increased morbidity and mortality. We aimed to investigate the associations between red, processed, and white meat consumption and the odds of developing kidney damage and DN in women. We enrolled 105 eligible women with DN and 105 controls (30–65 years). A validated and reliable food frequency questionnaire (FFQ) was used to evaluate the consumption of red, processed, and white meat. Biochemical variables and anthropometric measurements were assessed for all patients using pre-defined protocols. Binary logistic regression was conducted to examine possible associations. The results of the present study showed that there was a direct significant association between high consumption of red meat and processed meats and odds of microalbuminuria (red meat 2.30, 95% CI 1.25, 4.22; P-value = 0.007, processed meat: OR 2.16, 95% CI 1.18, 3.95; P-value = 0.01), severe albuminuria (red meat OR 3.25, 95% CI 1.38, 7.46; P-value = 0.007, processed meat: OR 2.35, 95% CI 1.01, 5.49; P-value = 0.04), BUN levels (red meat: OR 2.56, 95% CI 1.10, 5.93; P-value = 0.02, processed meat: OR 2.42, 95% CI 1.04, 5.62; P-value = 0.03), and DN (red meat 2.53, 95% CI 1.45, 4.42; P-value = 0.001, processed meat: OR 2.21; 95% CI 1.27, 3.85; P-value = 0.005). In summary, our study suggests that higher consumption of red and processed meat sources may be associated with microalbuminuria, severe albuminuria, higher BUN level, and higher odds of DN.

## Introduction

One of the most prevalent and severe side effects of diabetes mellitus is diabetic nephropathy (DN)^1^, which is linked to higher mortality and morbidity rates in diabetic individuals^2^. It is projected that as diabetes becomes more common, the prevalence of DN will also rise significantly if DN prevention does not improve promptly^3,4^.

Diabetes-related kidney damage, known as DN, is characterized by elevated blood pressure, decreased kidney function, and macroalbuminuria (urinary albumin excretion > 300 mg/day), among other symptoms^1,5^. "More than 50,000 diabetic people began therapy for end-stage renal disease (ESRD) in the US in 2014, compared to more than 40,000 in 2000" and "Consuming red and processed meat can increase oxidative stress and inflammation"^6^. Similarly, over 40% of Japanese diabetes patients in Asian nations have DN^7^. Ethnicity, family history, dyslipidemia, hypertension, insulin resistance, gestational diabetes, obesity, dyslipidemia, and high blood pressure are important risk factors for DN, some of which are controllable^8–10^. DN is the leading cause of ESRD in high-income countries, affecting around 25% of individuals with type 2 diabetes (T2D)^11^. Obesity, inactivity, and a poor diet are among the variables that may be contributing to the rise in the prevalence of type 2 diabetes mellitus (T2DM)^12,13^.

Diet composition significantly impacts insulin sensitivity and the chance of developing T2D^14^, which may help explain the considerable effect of dietary variables on the risk of T2D. Red meat, which includes beef, lamb, pork, and game, is an animal protein. It can be further divided into processed red meat (PRM) and unprocessed red meat (URM), depending on whether it has been altered to extend its shelf life through curing, smoking, salting, or adding chemical preservatives^15^. According to specific research, eating PRM or URM is linked favorably to developing T2D and insulin resistance^16–19^. Furthermore, it has been demonstrated that excessive consumption of red meat is strongly linked to higher levels of high-sensitivity C-reactive protein (hs-CRP)^20^. In contrast, consuming processed meat is linked to higher levels of interleukin 6 (IL-6) and lower tumor necrosis factor-alpha (TNF-) levels^21^. Consuming red and processed meat can increase oxidative stress and inflammation^20,22–25^. Oxidative stress is a typical byproduct of numerous metabolic pathways, including hyperglycemia itself, which are implicated in the pathogenesis of DN^26^. The main factor in the development of DN is an increase in reactive oxygen species (ROS) brought on by hyperglycemia^27^. Indeed, red meat is frequently discovered to contain nitrites, nitrates, heme iron, and advanced glycation end-products (AGEs)^28^. It is significant to note that individuals with ESRD have higher levels of circulatory AGEs, and dietary AGEs have been hypothesized to contribute to DM because of their oxidative capabilities^29^. The food sources acquired from the main food groups, particularly protein intake from different sources, are effective in eliciting insulin resistance and whole-body insulin sensitivity, and therefore inflammation, or directly in the development of inflammation^21,30–35^. However, some studies have found that fish and its byproducts can positively impact inflammatory markers^36–38^. According to one study, adding chicken to the usual diet (UD) instead of red meat decreased the urine albumin excretion rate (UAER) by 46%^39^, whilst withdrawing red meat from the diet reduced the UAER^40^. Also, a dietary pattern with white meats includes chicken and fish as the only meat protein source has been shown to decrease the glomerular filtration rate (GFR) in the hyper-filtering normoalbuminuric insulin-dependent diabetes mellitus (IDDM) sufferers^41^. Higher dietary intake of animal fat and two or more servings per week of red meat may increase the risk for microalbuminuria^42^; indeed, evidence from animal and human studies suggests that high protein consumption, especially animal protein, may accelerate the decline in GFR^43–45^. Urinary protein excretion, serum creatinine and uric acid levels, blood urea nitrogen (BUN) levels, and oxidative stress have all been documented to rise significantly in response to the elevated protein concentrations^46^.

Based on existing research, it seems that the consumption of red meat, processed meat, and white meat may be linked to the development of kidney damage and a condition known as Diabetic Nephropathy (DN). However, up to this point, no comprehensive study has been conducted to explore these connections. In light of this gap in the literature, we aimed to investigate the potential associations between the consumption of red meat, processed meat, and white meat, and the likelihood of women developing kidney damage and Diabetic Nephropathy.

## Results

### General characteristics of study population

This case–control study included 210 participants. The clinical characteristics of the participants are presented in Table 1. The mean and standard deviation (SD) for age, weight, and BMI of the individuals were 55.37 ± 7.07 years, 72.49 ± 12.71 kg, and 28.09 ± 4.59 kg/m^2^, respectively. In total, 50% of the participants had a history of nephropathy, and 22.4% had a history of cardiovascular disease (CVD).

**Table 1:** Characteristics of the study participants.

Quantitative variables		Mean	SD	Minimum	Maximum
Demographic characteristics and anthropometric					
Age (year)		55.37	7.07	35	65
Weight (kg)		72.49	12.71	48	117
Height (cm)		160.92	6.09	145	176
BMI (kg/m^2^)		28.09	4.59	17.6	45.7
Disease duration (month)		7.58	2.18	5	10
Blood pressure					
SBP (mmHg)		127.81	70.81	80	1120
DBP (mmHg)		81.45	12.48	50	120
Blood parameters					
FBS (mg/dL)		160.65	48.22	78	350
HbA1c (%)		8.34	1.38	5.7	12
TC (mg/dL)		180.27	35.63	106	285
TG (mg/dL)		164.75	61.82	59	460
HDL-c (mg/dL)		45.71	9.25	26	79
LDL-c (mg/dL)		100.73	31.17	32	190
BUN (mg/dL)		15.48	4.21	1	40
Cr (mg/dL)		0.89	0.16	0.60	1.60
Alb (mg/dL)		11.39	10.14	2	100
ACR (mg/g)		125.43	133.96	9	534
GFR (mL/min)		98.52	27.7	11.11	175
Qualitative variables		N	%		
Nephropathy history					
Yes		105	50	–	–
No		105	50	–	–
CVD history					
Yes		47	22.4	–	–
No		163	77.6	–	–
Drugs user					
ARB					
Yes		105	50	–	–
No		105	50	–	–
ACIE					
Yes		65	31	–	–
No		145	69	–	–
Beta-blocker					
Yes		38	18.1	–	–
No		171	81.4	–	–
Metformin					
Yes		208	99	–	–
No		2	1	–	–
Sulfonylurea					
Yes		133	63.3	–	–
No		77	36.7	–	–
Insulin					
Yes		61	29	–	–
No		149	71	–	–

Significant values are in bold.

*BMI* body mass index, *SBP* systolic blood pressure, *DBP* diastolic blood pressure, *FBS* fasting blood sugar, *HbA1c* glycosylated hemoglobin type A1c, *TC* total cholesterol, *TG* triglyceride, *HDL-c* high-density lipoprotein, *LDL-c* low-density lipoprotein, *BUN* blood urea nitrogen, *Cr* creatinine, *CVD* cardiovascular disease, *ARB* angiotensin receptor blockers, *ACIE* angiotensin-converting enzyme inhibitors.

All data are presented as mean and SD or N and %.

### General characteristics and dietary intakes of participants according to intakes of red, white, and processed meat

The general characteristics of the study among participants with low and high red, white, and processed meat intake are shown in Table 2. In the case group, a positive and significant difference was observed between the levels of FBS (P-value = 0.02) and Cr (P-value = 0.04) and the amount of red meat intake. No significant differences were found in other variables (P-value > 0.05). In the control group, a significant difference in ACIE intake was observed (P-value = 0.04). Greater red meat intake was associated with higher FBS (P-value = 0.05), TC (P-value = 0.06), LDL-c (P-value = 0.03), BUN (P-value = 0.05), and albumin (P-value = 0.004) levels. A significant inverse difference was observed between DBP (P-value = 0.007) and TC (P-value = 0.02) with the intake of white meat in the subject group. More consumption of white meat was associated with lower levels of DBP and TC in the case group, whilst in the control group, higher white meat intake was associated with lower HbA1c (P-value = 0.03), TG (P-value = 0.03), and albumin (P-value = 0.002). In the case group, higher processed meat intake was associated with higher FBS (P-value = 0.05) and Cr (P-value = 0.02). In the control group, a positive and significant difference was found between the level of FBS and the amount of processed meat consumption (P-value = 0.03).

**Table 2:** General characteristics of study population according on intake of red meat, white meat, and processed meat (N = 210).

	Red meat												White meat										Processed meat					
Characteristics	Case				P-value			Control		P-value			Case		P-value		Control				P-value		Case		P-value	Control		P-value
Quantitative variables	Low (≤ 32.56) (N = 41)			High (> 32.56) (N = 64)				Low (≤ 32.56) (N = 65)	High (> 32.56) (N = 40)				Low (≤ 17.79) (N = 73)	High (> 17.79) (N = 32)			Low (≤ 17.79) (N = 32)		High (> 17.79) (N = 72)				Low (≤ 13.35) (N = 41)	High (> 13.35) (N = 63)		Low (≤ 13.35) (N = 62)	High (> 13.35) (N = 43)	
Age (year)	55.73 ± 7.42			55.08 ± 6.83	0.64			56.14 ± 6.38	54.23 ± 8.17	0.18			55.32 ± 6.96	55.38 ± 7.31	0.96		54.63 ± 8.24		55.75 ± 6.63		0.45		56.32 ± 5.99	54.59 ± 7.61	0.22	55.63 ± 6.76	55.09 ± 7.71	0.71
Weight (kg)	71.58 ± 11.31			74.56 ± 15.19	0.28			71.84 ± 10.94	71.18 ± 12.47	0.77			73.45 ± 15.10	73.28 ± 10.54	0.93		72.33 ± 11.10		71.26 ± 11.72		0.79		71.86 ± 12.67	74.26 ± 14.61	0.39	71.25 ± 10.55	72.06 ± 12.85	0.72
Height(cm)	160.34 ± 6.39			160.89 ± 6.25	0.66			160.28 ± 6.37	162.63 ± 4.80	**0.04**			160.48 ± 5.91	161.13 ± 7.14	0.57		163.16 ± 5.23		160.30 ± 6.01		0.09		159.98 ± 6.50	161.24 ± 6.14	0.31	160.06 ± 6.32	162.77 ± 4.90	**0.02**
BMI (kg/m^2^)	27.79 ± 4.22			29.26 ± 4.99	0.12			27.93 ± 4.03	26.82 ± 4.88	0.21			28.92 ± 4.98	28.13 ± 4.15	0.52		27.20 ± 4.63		27.64 ± 4.30		0.73		28.006 ± 4.94	29.03 ± 4.57	0.28	27.80 ± 4.04	27.08 ± 4.86	0.41
SBP (mmHg)	127.61 ± 16.79			125.94 ± 17.66	0.63			133.92 ± 125.19	121.10 ± 15.70	0.52			126.27 ± 16.83	127.31 ± 18.47	0.80		116.78 ± 16.02		134.41 ± 117.96		0.30		125.76 ± 17.63	127.24 ± 17.26	0.67	133.81 ± 128.23	122.16 ± 15.62	0.55
DBP (mmHg)	82.02 ± 10.89			83.30 ± 14.37	0.62			80.48 ± 11.93	79.48 ± 11.58	0.67			84.90 ± 13.61	78 ± 10.49	**0.007**		78.56 ± 11.51		80.77 ± 11.88		0.51		81.90 ± 12.45	83.75 ± 13.32	0.48	79.81 ± 12.13	80.51 ± 11.32	0.76
FBS (mg/dL)	153.37 ± 31.59			175.91 ± 58.26	**0.02**			147.62 ± 46.23	164.88 ± 41.35	**0.05**			171.37 ± 55.17	157.37 ± 37.19	0.30		157.59 ± 36.48		152.70 ± 48.46		0.54		154.46 ± 34.22	174.08 ± 57.32	**0.05**	146.61 ± 42.75	165.12 ± 46.46	**0.03**
HbA1c (%)	8.37 ± 1.28			8.84 ± 1.46	0.09			7.89 ± 1.37	8.25 ± 1.11	0.17			8.73 ± 1.35	8.49 ± 1.52	0.63		8.37 ± 1.45		7.87 ± 1.19		**0.03**		8.59 ± 1.36	8.65 ± 1.41	0.82	7.88 ± 1.32	8.23 ± 1.22	0.17
TC (mg/dL)	189.15 ± 38.91			182.59 ± 37.68	0.39			170.86 ± 31.50	182.73 ± 32.92	**0.06**			190.67 ± 36.57	172.56 ± 39.14	**0.02**		176.53 ± 27.80		174.88 ± 34.41		0.92		187.07 ± 40.55	184.65 ± 36.57	0.75	173.74 ± 31.54	177.74 ± 33.87	0.53
TG (mg/dL)	167.05 ± 59.65			167.39 ± 69.73	0.97			157.68 ± 60.82	169.68 ± 52.71	0.30			171.40 ± 55.68	157.81 ± 84.49	0.34		178.63 ± 47.84		155.07 ± 60.71		**0.03**		169.93 ± 74.62	166.78 ± 59.48	0.81	160.13 ± 60.32	165.30 ± 54.79	0.65
HDL-c (mg/dL)	44.12 ± 9.49			45.64 ± 9.12	0.41			47.42 ± 8.91	44.68 ± 9.64	0.14			44.99 ± 9.24	45.19 ± 9.43	0.89		44.62 ± 7.37		47.14 ± 9.79		0.19		45.49 ± 8.50	44.37 ± 9.29	0.53	47.56 ± 9.13	44.65 ± 9.24	0.11
LDL-c (mg/dL)	107.29 ± 30.95			106.58 ± 32.52	0.91			89.80 ± 27.45	102.40 ± 31.27	**0.03**			110.34 ± 30.52	98.91 ± 33.59	0.09		100.53 ± 28.97		92 ± 29.50		0.18		109.44 ± 28.79	106.08 ± 33.18	0.59	93.45 ± 29.50	96.26 ± 29.69	0.63
BUN (mg/dL)	15.83 ± 5.38			15.77 ± 3.96	0.94			14.60 ± 3.23	16.10 ± 4.59	**0.05**			15.57 ± 3.86	16.31 ± 5.84	0.40		15.75 ± 4.15		14.92 ± 3.72		0.46		15.75 ± 5.46	15.87 ± 3.90	0.90	14.81 ± 3.79	15.70 ± 3.93	0.24
Cr (mg/dL)	0.88 ± 0.14		0.94 ± 0.16		**0.04**			0.86 ± 0.17	0.88 ± 0.16	0.50			0.93 ± 0.15	0.90 ± 0.17	0.44		0.88 ± 0.15		0.86 ± 0.17		0.59		0.87 ± 0.14	0.95 ± 0.16	**0.02**	0.88 ± 0.18	0.86 ± 0.15	0.56
Albumin (mg/dL)	14.41 ± 10.35			14.39 ± 12.93	0.99			6.90 ± 6.14	10.78 ± 7.09	**0.004**			13.90 ± 7.85	15.53 ± 18.24	0.44		11.09 ± 6.61		7.18 ± 6.52		**0.002**		11.98 ± 7.62	16.02 ± 13.96	0.09	8.15 ± 6.66	8.70 ± 6.97	0.68
ACR (mg/g)	220.07 ± 132.45			239.97 ± 100.92	0.38			18.94 ± 5.95	18.22 ± 5.91	0.55			241.21 ± 101.62	211.63 ± 138	0.22		20.29 ± 5.61		17.95 ± 5.95		0.05		226.45 ± 126.83	232.82 ± 103.95	0.78	19.31 ± 5.73	17.74 ± 6.12	0.18
GFR (mL/min)	98.06 ± 25.57			95.18 ± 26.19	0.58			101.11 ± 32.4	100.11 ± 32.3	0.86			95.07 ± 25.92	99.12 ± 25.93	0.46		101.07 ± 30.6		100.59 ± 29.3		0.93		97.82 ± 25.31	95.04 ± 26.47	0.59	99.11 ± 27.45	103.09 ± 32.16	0.49
Disease duration (month)	7.32 ± 2.21			7.79 ± 2.19	0.29			7.43 ± 2.17	7.76 ± 2.16	0.44			7.59 ± 2.18	7.62 ± 2.28	0.91		7.33 ± 2.11		7.66 ± 2.19		0.56		8.12 ± 2.10	7.23 ± 2.21	**0.04**	7.85 ± 2.14	7.13 ± 2.14	0.09
Qualitative variables																												
Physical activity																												
Low	15 (48.4)			16 (51.6)	0.50			23 (62.2)	14 (37.8)	0.31			21 (67.7)	10 (32.3)	0.93		8 (21.6)		29 (78.4)		0.10		10 (32.3)	21 (67.7)	0.61	21 (56.8)	16 (43.2)	0.85
Moderate	13 (31)			29 (69)				15 (53.6)	13 (46.4)				29 (69)	13 (31)			7 (25)		21 (75)				17 (41.5)	25 (58.5)		16 (57.1)	12 (42.9)	
High	13 (40.6)			19 (59.4)				27 (67.5)	13 (32.5)				23 (71.9)	9 (28.1)			17 (42.5)		23 (57.5)				14 (43.8)	18 (56.3)		25 (62.5)	15 (37.5)	
Nephropathy history																												
Yes	41 (39.4)			63 (60.6)	0.42			1 (100)	0 (0)	0.43			73 (70.2)	31 (29.8)	0.12		0 (0)		1 (100)		0.50		41 (39.8)	62 (60.2)	0.41	0 (0)	1 (100)	0.22
No	0 (0)			1 (100)				64 (61.5)	40 (38.5)				0 (0)	1 (100)			32 (30.8)		72 (69.2)				0 (0)	1 (100)		62 (59.6)	42 (40.4)	
CVD history																												
Yes	11 (45.8)			13 (54.2)	0.27			12 (52.2)	11 (47.8)	0.43			15 (62.5)	9 (37.5)	0.39		6 (26.1)		17 (73.9)		0.60		9 (39.1)	14 (60.9)	0.97	12 (52.2)	11 (47.8)	0.44
No	30 (37)			51 (63)				53 (64.6)	29 (35.4)				58 (71.6)	23 (28.4)			26 (31.7)		56 (68.3)				32 (39.5)	49 (60.5)		50 (61)	32 (39)	
Drugs user																												
ARB																												
Yes	24 (40)			36 (60)	0.81			25 (55.6)	20 (44.4)	0.24			39 (65)	21 (35)	0.24		12 (26.7)		33 (73.3)		0.46		23 (39)	36 (61)		25 (55.6)	20 (44.4)	
No	17 (37.8)			28 (62.2)				40 (66.7)	20 (33.3)				34 (75.6)	11 (24.4)			20 (33.3)		40 (66.7)				18 (40)	27 (60)	0.91	37 (61.7)	23 (38.3)	0.52
ACIE																												
Yes	20 (45.5)			24 (54.5)	0.24			9 (42.9)	12 (57.1)	**0.04**			30 (68.2)	14 (31.8)	0.80		6 (28.6)		15 (71.4)		0.83		17 (39.5)	26 (60.5)	0.98	10 (47.6)	11 (52.4)	0.23
No	21 (34.4)			40 (65.6)				56 (66.7)	28 (33.3)				43 (70.5)	18 (29.5)			26 (31)		58 (69)				24 (39.3)	37 (60.7)		52 (61.9)	32 (38.1)	
Beta-blocker																												
Yes	8 (40)			12 (60)	0.44			9 (50)	9 (50)	0.25			15 (75)	5 (25)	0.27		3 (16.7)		15 (83.3)		0.16		8 (42.1)	11 (57.9)	0.43	10 (55.6)	8 (44.4)	0.74
No	32 (38.1)			52 (61.9)				56 (64.4)	31 (35.6)				58 (69)	26 (31)			29 (33.3)		58 (66.7)				32 (38.1)	52 (61.9)		52 (59.8)	35 (40.2)	
Metformin																												
Yes	41 (39.4)			63 (60.6)	0.20			65 (62.5)	39 (37.5)	0.42			73 (70.2)	31 (29.8)	0.12		32 (30.8)		72 (69.2)		0.50		41 (39.8)	62 (60.2)	0.41	62 (59.6)	42 (40.4)	0.22
No	0 (0)			1 (100)				0 (0)	1 (100)				0 (0)	1 (100)			0 (0)		1 (100)				0 (0)	1 (100)		0 (0)	1 (100)	
Sulfonylurea																												
Yes	29 (40.8)			42 (59.2)	0.58			38 (61.3)	24 (38.7)	0.87			48 (67.6)	23 (32.4)	0.53		21 (33.9)		41 (66.1)		0.36		29 (41.4)	41 (58.6)	0.54	35 (56.5)	27 (43.5)	0.51
No	12 (35.3)			22 (64.7)				27 (62.8)	16 (37.2)				25 (73.5)	9 (26.5)			11 (25.6)		32 (74.4)				12 (35.3)	22 (64.7)		27 (62.8)	16 (37.2)	
Insulin																												
Yes	7 (26.9)			19 (73.1)	0.14			21 (60)	14 (40)	0.77			21 (80.8)	5 (19.2)	0.15		12 (34.3)		23 (65.7)		0.54		9 (34.6)	17 (65.4)	0.56	23 (65.7)	12 (34.3)	0.32
No	34 (43)			45 (57)				44 (62.9)	26 (37.1)				52 (65.8)	27 (34.2)			20 (28.6)		50 (71.4)				32 (41)	46 (59)		39 (55.7)	31 (44.3)	

Significant values are in bold.

*BMI* body mass index, *SBP* systolic blood pressure, *DBP* diastolic blood pressure, *FBS* fasting blood sugar, *HbA1c* glycosylated hemoglobin type A1c, *TC* total cholesterol, *TG* triglyceride, *HDL-c* high-density lipoprotein, *LDL-c* low-density lipoprotein, *BUN* blood urea nitrogen, *Cr* creatinine, *CVD* cardiovascular disease, *ARB* angiotensin receptor blockers, *ACIE* angiotensin-converting enzyme inhibitors.

Quantitative variables are represented as means ± SD.

Qualitative variables: N (%).

P-values < 0.05 were considered significant.

The dietary intake of the study participants, based on the mean consumption of red meat, white meat, and processed meat, is presented in Table 3. After adjustment for confounders (age and total energy intake), a significant positive difference was observed between the mean values of total fat, MUFA, oleic acid, B5, vitamin K, vitamin E, vitamin A, sodium, calcium, glucose, galactose, fructose, and sucrose with the intake of red meat. There was also a significant inverse difference between the mean values of vitamin E, B5, glucose, and fructose with the intake of red meat (P-value < 0.05). In the adjusted model, there was a significant direct difference between the mean values of protein, oleic acid, B5, vitamin E, potassium, calcium, phosphor, magnesium, and sucrose with the consumption of white meat, except for oleic acid (P-value < 0.05). In the adjusted model, a significant direct difference was observed between the mean values of MUFA, oleic acid, B6, vitamin K, sodium, calcium, magnesium, galactose, and sucrose with the intake of processed meat, except for magnesium (P-value < 0.05).

**Table 3:** Dietary intakes of study population among intakes of red meat, white meat, and processed meat (N = 210).

Variables	Red meat						White meat				Processed meat			
	Low (≤ 32.56)			High (> 32.56)	P-value	P-value*	Low (≤ 17.79)	High (> 17.79)	P-value	P-value*	Low (≤ 13.35)	High (> 13.35)	P-value	P-value*
	Mean ± SD			Mean ± SD			Mean ± SD	Mean ± SD			Mean ± SD	Mean ± SD		
Macronutrients														
Carbohydrates (g/day)	243.45 ± 65.67			259.15 ± 44.14	**0.04**	0.10	249.30 ± 39.94	252.78 ± 69.22	0.66	0.47	246.50 ± 64.59	254.67 ± 46.50	0.30	0.98
Protein (g/day)	46.08 ± 9.73			47.99 ± 8.77	0.14	0.11	45.83 ± 6.91	48.14 ± 11.04	0.07	**0.001**	46.98 ± 10.15	46.86 ± 8.26	0.93	**0.03**
Total fat (g/day)	31.007 ± 6.03			34.61 ± 9.19	**0.001**	**0.03**	33.15 ± 8.28	32.37 ± 7.56	0.47	0.21	32.03 ± 7.05	33.32 ± 8.55	0.24	0.55
CHOL (g/day)	6.91 ± 9.10			6.63 ± 7.34	0.80	0.08	5.95 ± 4.26	7.57 ± 10.78	0.15	0.14	6.88 ± 8.72	6.66 ± 7.88	0.85	0.42
Sub types of fatty acid														
Saturated fat (g/day)	5.84 ± 1.47			6.50 ± 1.73	**0.003**	0.09	6.25 ± 1.57	6.08 ± 1.69	0.46	0.17	6.16 ± 1.66	6.13 ± 1.58	0.88	0.20
MUFA (g/day)	10.13 ± 2.14			11.89 ± 3.66	** < 0.001**	**0.001**	11.24 ± 3.30	10.73 ± 2.89	0.24	**0.06**	10.51 ± 2.62	11.40 ± 3.43	**0.03**	**0.06**
PUFA (g/day)	10.15 ± 2.40			10.90 ± 2.31	**0.02**	0.25	10.42 ± 1.95	10.60 ± 2.74	0.59	0.65	10.44 ± 2.71	10.53 ± 1.95	0.79	0.74
Oleic acid (g/day)	9.79 ± 2.02			11.57 ± 3.52	** < 0.001**	** < 0.001**	10.94 ± 3.20	10.38 ± 2.74	0.17	**0.03**	10.12 ± 2.43	11.13 ± 3.33	**0.01**	**0.02**
Linoleic acid (g/day)	9.09 ± 2.27			9.75 ± 2.05	**0.03**	0.30	9.31 ± 1.71	9.50 ± 2.57	0.53	0.58	9.36 ± 2.53	9.40 ± 1.71	0.89	0.63
Linolenic acid (g/day)	0.88 ± 0.29			0.95 ± 0.28	**0.06**	0.16	0.92 ± 0.25	0.91 ± 0.32	0.82	0.74	0.89 ± 0.32	0.93 ± 0.25	0.33	0.42
Total fiber (g/day)	37.56 ± 10.04			39.42 ± 6.93	0.12	0.84	38.27 ± 7.68	38.65 ± 9.61	0.75	0.80	38.02 ± 10.07	38.92 ± 7.13	0.46	0.88
Micronutrients														
Thiamin (mg/day)	1.62 ± 0.33			1.76 ± 0.31	**0.002**	0.12	1.69 ± 0.27	1.68 ± 0.38	0.86	0.65	1.65 ± 0.34	1.71 ± 0.31	0.18	0.56
Riboflavin (mg/day)	0.93 ± 0.17			1.01 ± 0.20	**0.003**	0.16	0.98 ± 0.18	0.96 ± 0.20	0.57	0.29	0.94 ± 0.17	0.98 ± 0.20	0.15	0.55
Niacin (mg/day)	15.41 ± 2.89			16.74 ± 3.05	**0.002**	0.09	16.16 ± 2.79	16.96 ± 3.26	0.63	0.32	15.66 ± 3.001	16.42 ± 3.03	0.07	0.20
Pantothenic acid (mg/day)	2.49 ± 0.66			2.42 ± 0.66	0.45	**0.009**	2.35 ± 0.65	2.56 ± 0.66	**0.02**	**0.009**	2.46 ± 0.69	2.46 ± 0.64	0.96	0.46
Pyridoxine (mg/day)	0.74 ± 0.13			0.79 ± 0.14	**0.005**	**0.06**	0.75 ± 0.13	0.77 ± 0.14	0.30	0.31	0.73 ± 0.14	0.79 ± 0.13	**0.004**	**0.007**
Folic acid (mg/day)	376.24 ± 100.98			392.12 ± 91.50	0.23	0.44	380.15 ± 76.35	387.60 ± 112.95	0.58	0.55	384.23 ± 103.67	383.06 ± 89.68	0.93	0.23
Cobalamin (mg/day)	0.14 ± 0.15			0.15 ± 0.13	0.65	0.35	0.13 ± 0.08	0.15 ± 0.19	0.50	0.54	0.14 ± 0.15	0.14 ± 0.14	0.85	0.70
Vitamin K (mg/day)	12.0005 ± 3.77			14.25 ± 5.91	**0.001**	**0.02**	13.50 ± 5.51	12.69 ± 4.54	0.25	0.14	12.21 ± 4.54	13.95 ± 5.40	**0.01**	**0.02**
Vitamin E (mg/day)	4.12 ± 1.84			4.01 ± 1.007	0.61	**0.003**	3.82 ± 0.96	4.30 ± 1.84	**0.02**	**0.004**	4.14 ± 1.83	3.99 ± 1.05	0.47	**0.06**
Vitamin C (mg/day)	10.44 ± 6.53			10.64 ± 4.75	0.79	0.45	10.07 ± 4.15	11.004 ± 6.90	0.24	0.25	10.34 ± 6.59	10.73 ± 4.74	0.62	0.91
Biotin (mg/day)	17.79 ± 5.58			17.32 ± 5.49	0.54	0.32	16.84 ± 5.53	18.27 ± 5.47	**0.06**	**0.06**	17.66 ± 5.72	17.45 ± 5.38	0.78	0.63
Retinol (mg/day)	19.40 ± 12.41			26.21 ± 13.47	** < 0.001**	**0.001**	24.002 ± 11.58	21.46 ± 14.81	0.17	0.13	19.59 ± 12.56	25.75 ± 13.50	**0.001**	**0.001**
Sodium (mg/day)	3248.88 ± 919.74			3774.88 ± 1010.89	** < 0.001**	**0.001**	3613.09 ± 1019.12	3399.50 ± 970.80	0.12	0.11	3323.31 ± 876.71	3680.92 ± 1084.99	**0.01**	**0.02**
Potassium (mg/day)	1703.52 ± 479.11			1708.13 ± 278.77	0.93	**0.05**	1635.36 ± 263.71	1773.48 ± 478.99	**0.01**	**0.002**	1718.43 ± 489.13	1685.54 ± 258.75	0.54	0.10
Calcium (mg/day)	381.23 ± 68.31			426.04 ± 71.72	** < 0.001**	** < 0.001**	409.63 ± 61.49	396.59 ± 82.96	0.20	**0.01**	389.62 ± 69.68	415.29 ± 74.39	**0.01**	**0.007**
Iron (mg/day)	14.50 ± 2.51			15.30 ± 2.26	**0.01**	0.87	14.69 ± 1.99	15.07 ± 2.76	0.26	**0.05**	14.73 ± 2.61	15.004 ± 2.18	0.42	0.63
Phosphor (mg/day)	886.54 ± 172.74			924.48 ± 155.59	0.10	0.79	882.96 ± 132.16	926.11 ± 190.17	**0.06**	**0.01**	896.88 ± 184.68	910.26 ± 141.79	0.56	0.75
Magnesium (mg/day)	350.67 ± 87.89			359.57 ± 60.24	0.40	0.48	342.33 ± 52.76	367.17 ± 91.17	**0.01**	**0.007**	360.23 ± 90.39	347.72 ± 53.75	0.23	**0.02**
Chromium (mg/day)	0.21 ± 0.07			0.22 ± 0.07	0.55	0.89	0.20 ± 0.06	0.22 ± 0.07	**0.06**	0.07	0.21 ± 0.07	0.21 ± 0.07	0.75	0.60
Glucose (g/day)	12.94 ± 14.89			11.22 ± 3.0005	0.25	**0.003**	10.83 ± 3.04	13.34 ± 14.89	0.09	0.08	12.76 ± 14.60	11.43 ± 4.96	0.38	0.10
Galactose (g/day)	0.11 ± 0.08			0.17 ± 0.08	** < 0.001**	** < 0.001**	0.15 ± 0.09	0.13 ± 0.08	0.11	0.08	0.12 ± 0.08	0.16 ± 0.08	**0.001**	**0.003**
Fructose (g/day)	14.19 ± 15.76			12.28 ± 3.20	0.23	**0.002**	11.83 ± 3.28	14.64 ± 15.76	0.08	**0.06**	14.02 ± 15.45	12.48 ± 5.29	0.34	0.08
Sucrose (g/day)	10.80 ± 7.17			15.30 ± 7.08	** < 0.001**	** < 0.001**	14.96 ± 7.67	11.08 ± 6.75	** < 0.001**	** < 0.001**	10.16 ± 5.73	15.68 ± 7.88	** < 0.001**	** < 0.001**

Significant values are in bold.

*CHOL* cholesterol, *MUFA* monounsaturated fatty acid, *PUFA* polyunsaturated fatty acid.

All data are presented as mean ± SD.

P-value * is obtained by adjusting the variables of age and total energy intake.

P-values < 0.05 were considered significant.

### The association between red, white, and processed meat and odds of DN, ACR, GFR and increase BUN

**Figure 1:**
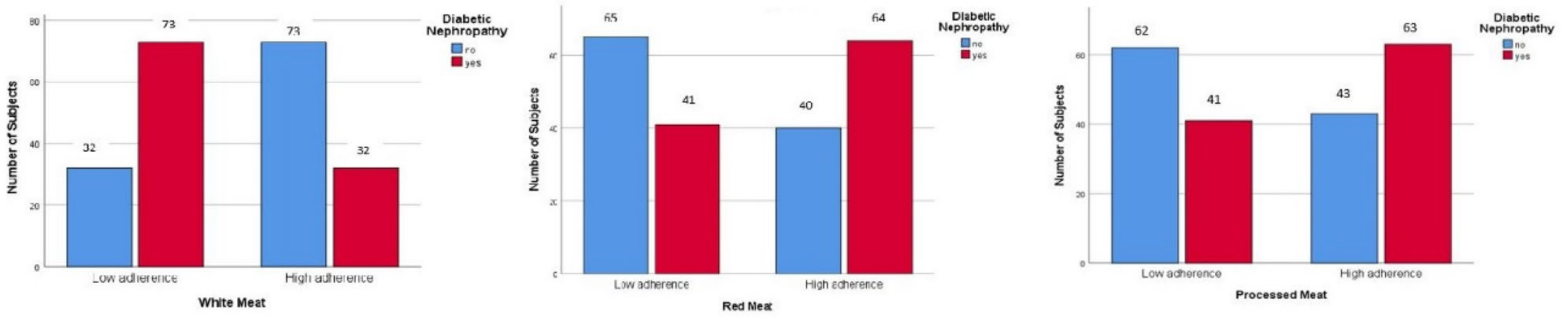
Number subjects of DN among intake of red meat, white meat, and processed meat.

Binary logistic regression was used, in both crude and adjusted models (adjustment for confounders such as age, energy intake, albumin, hemoglobin, physical activity, CVD, and disease duration), to investigate the association between consumption of different types of meat with the odds of DN (Table 4). There was a significant direct relationship between high consumption of red meat (OR 2.53, 95% CI 1.45, 4.42; P-value = 0.001) and odds of DN, which remained significant after adjusting for confounding variables (OR 2.62, 95% CI 1.39, 4.93; P-value = 0.003). A higher intake of white meat was associated with a reduction in the risks of DM by 81% in the crude model (OR 0.19; 95% CI 0.10, 0.34; P-value 0.001), remained significant after adjusting for confounding variables (OR 0.20 95% CI 0.10, 0.40; P-value < 0.001), equating to an 80% reduction in odds of DN. The odds of DN in individuals with a high intake of processed meats is 2.21 times higher than the reference group (OR 2.21; 95% CI 1.27, 3.85; P-value = 0.005), decreased to 2.19 after adjustment (OR 2.19; 95% CI 1.17, 4.12; P-value = 0.01) (Fig. 1).

**Table 4:** Association between intake of red meat, white meat, and processed meat and odds of DN and developing kidney damage (N = 210).

Variables	Red meat						White meat						Processed meat					
	Crude model			Adjust model			Crude model			Adjust model			Crude model			Adjust model		
	OR	0.95% CI	P-value	OR	0.95% CI	P-value	OR	0.95% CI	P-value	OR	0.95% CI	P-value	OR	0.95%CI	P-value	OR	0.95%CI	P-value
Nephropathy																		
Control	Reference						Reference						Reference					
Case	2.53	1.45–4.42	**0.001**	2.62	1.39–4.93	**0.003**	0.19	0.10–0.34	** < 0.001**	0.20	0.10–0.40	** < 0.001**	2.21	1.27–3.85	**0.005**	**2.19**	**1.17–4.12**	**0.01**
ACR																		
Normal	Reference						Reference						Reference					
Microalbuminuria	2.30	1.25–4.22	**0.007**	2.46	1.26–4.83	**0.008**	0.21	0.11–0.41	** < 0.001**	0.23	0.11–0.47	** < 0.001**	2.16	1.18–3.95	**0.01**	2.19	1.12–4.27	0.12
Severely albuminuria	3.25	1.38–7.46	**0.007**	3.22	1.20–8.59	**0.02**	0.13	0.05–0.34	** < 0.001**	0.13	0.04–0.39	** < 0.001**	2.35	1.01–5.49	**0.04**	2.16	0.81–5.76	**0.02**
GFR																		
Normal	Reference						Reference						Reference					
Mild to severe decrease	1.53	0.88–2.65	0.13	2.87	1.10–7.51	**0.03**	0.62	0.35–1.08	0.09	1.12	0.46–2.71	0.79	1.30	0.75–2.26	0.34	2.55	1.02–6.53	**0.05**
BUN																		
Normal	Reference						Reference						Reference					
Mild to severe increase	2.56	1.10–5.93	**0.02**	1.84	0.94–3.61	0.07	0.92	0.42–2.02	0.84	0.58	0.29–1.16	0.12	2.42	1.04–5.62	**0.03**	1.66	0.85–3.25	0.13

Significant values are in bold.

*DN* diabetic nephropathy, *ACR* albumin-to-creatinine ratio, *GRR* glomerular filtration rate, *BUN* blood urea nitrogen.

All data are presented as OR and 95% Cl by binary logistic regression.

P-value for adjustment model: based on age, energy intake, albumin, hemoglobin, physical activity, CVD, and disease duration.

P-values < 0.05 were considered significant.

**Figure 2:**
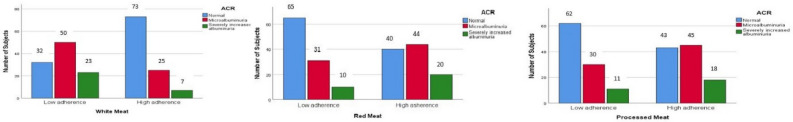
Levels of ACR among intake of red meat, white meat, and processed meat.

There was a direct significant relationship between high consumption of red meat and odds of microalbuminuria (OR 2.30, 95% CI 1.25, 4.22; P-value = 0.007) and severe albuminuria (OR 3.25, 95% CI 1.38, 7.46; P-value = 0.007). Also, this significant association remained even after adjustment for potential confounding variables. The occurrence of microalbuminuria and severe albuminuria were 2.46 and 3.22 times greater, respectively, in high consumption of red meat compared to low intake (Table 4). In the crude model, there was an inverse association between higher consumption of white meat compared with low intake associated with odds of microalbuminuria (OR 0.21, 95% CI 0.11, 0.41; P-value < 0.001) and severe albuminuria (OR 0.13, 95% CI 0.05, 0.34; P-value < 0.001). High consumption of white meat, compared with low intake, was associated with lower odds of microalbuminuria (OR 0.23, 95% CI 0.11, 0.47; P-value < 0.001) and severe albuminuria (OR 0.13, 95% CI 0.04, 0.39; P-value < 0.001). In the crude model, there was a direct significant association between higher intake of processed meats, compared to low intake, and higher odds of microalbuminuria (OR 2.16, 95% CI 1.18, 3.95; P-value = 0.01) and severe albuminuria (OR 2.35, 95% CI 1.01, 5.49; P-value = 0.04) in participants. In the adjusted model, the odds of severe albuminuria were 2.16 times greater when participants consumed more processed meats (OR 2.16, 95% CI 0.81, 5.76; P-value = 0.02) (Table 4, Fig. 2).

**Figure 3:**
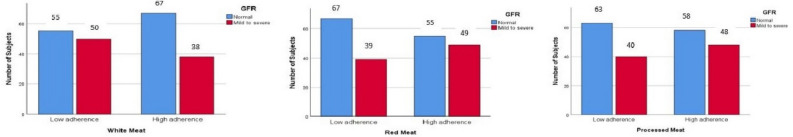
Levels of GFR among intake of red meat, white meat, and processed meat.

In the adjusted model, higher consumption of red meat was associated with higher odds of mild to severe GFR decrease (OR 2.87; 95% CI 1.10, 7.51; P-value = 0.03). And also, the odds of mild to severe GFR decrease were 2.55 times higher when participants consumed more processed (OR 2.55, 95% CI 1.02, 6.53; P-value = 0.05) (Table 4, Fig. 3).

**Figure 4:**
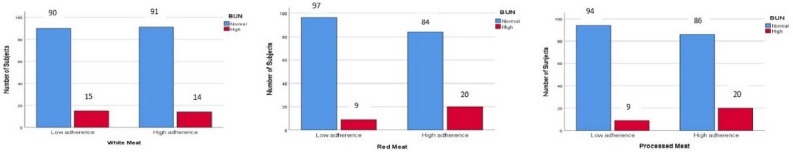
Levels of BUN among intake of red meat, white meat, and processed meat.

In the crude model, there was a direct significant association between consumption of red meat and greater odds of mild to severe increased BUN (OR 2.56, 95% CI 1.10, 5.93; P-value = 0.02). Patients with higher consumption of processed meats had 2.42 times higher odds of increased BUN (OR 2.42, 95% CI 1.04, 5.62; P-value = 0.03) in the crude model (Table 4, Fig. 4).

## Discussion

To the best of the authors' knowledge, this study is the first to investigate the relationship between the prevalence of DN, renal function indicators, and the consumption of various types of meat. The results of the current case–control study demonstrated that increased consumption of red and processed meat sources was associated with microalbuminuria, severe albuminuria, higher BUN levels, and higher odds ratios for developing DN. In the adjusted model, an inverse relationship between GFR and the consumption of red and processed meat was observed. Individuals with high consumption of white meat sources had lower odds ratios for microalbuminuria, severe albuminuria, and DN.

The results of the current case–control study align with previous research on the relationship between the consumption of various types of meat and renal function in a healthy population. Notably, a study involving 3000 women found that a dietary intake high in animal fat and two or more servings of red meat was associated with microalbuminuria^42^. In a cross-sectional study with a population of 19,000 people, the Western dietary style was linked to hyper-albuminuria due to its high consumption of saturated fatty acids. However, polyunsaturated fatty acids (PUFAs) and trans fatty acids were neither associated with hyper-albuminuria nor with the estimated glomerular filtration rate (eGFR)^47^. A previous study reported that high scores for the Western dietary pattern were correlated with microalbuminuria and a rapid decline in eGFR^48^. Furthermore, a higher intake of long-chain omega-3 PUFAs and fish was linked to a decreased incidence of chronic kidney disease (CKD) in those over 50, although this connection was not found with the consumption of total omega-3 and omega-6 PUFAs^49^.

The findings of the current case–control study indicated that higher consumption of red meat was associated with increased odds of DN, which aligns with cohort studies conducted in healthy populations. The frequency of chronic kidney disease (CKD) in a healthy population showed inconsistent correlations with processed meat^50,51^ and fish intake^50,52^. In the present investigation, the prevalence of DN was positively correlated with consuming processed meat and negatively correlated with white meat intake. Following healthy eating patterns in diabetic patients has been shown to help maintain optimal kidney function^53–57^. Studies on the amount of protein necessary to maintain renal function have produced conflicting results. Both the quantity and source of dietary protein have been associated with renal dysfunction and tissue damage in both healthy and diabetic adult^58^. Moreover, several studies evaluating the effects of low protein diets (LPD) in animal models have found that LPD has reno-protective benefits in the presence of renal disorders^59,60^. In animal models of type 2 diabetes with advanced diabetic nephropathy, LPD has been effective in restoring autophagy by suppressing the mechanistic target of rapamycin complex (mTORC1)^61^.

However, despite the beneficial effects of LPD reported in animal models and earlier research^40,62^ a meta-analysis found no advantage of a low-protein diet over a higher protein diet for diabetic nephropathy, improving glomerular filtration rate (GFR), or reducing proteinuria^63^. An observational study of 6213 subjects with type 2 diabetes found no clear benefit in renal parameters from a low-protein diet^64^. According to randomized controlled trials (RCTs), the type of protein is just as crucial as the quantity of protein^40,65^. Similar to LPD, diet protein based on chicken reduced and elevated blood levels of UAER and PUFAs, respectively, in research done on T2D patients with macroalbuminuria^40^. The results in type 1 diabetes patients were consistent with the study by Mello et al.^62^. The current case–control study's findings align with previous research that suggested a connection between increased consumption of white protein sources and a reduced risk of DN. Chicken has been shown to reduce urinary albumin excretion rate (UAER) by 36% in type 2 diabetes (T2D) patients with microalbuminuria compared to a typical diet^39^. Additionally, chicken and a low-protein diet (LPD) caused total cholesterol (TC), low-density lipoprotein (LDL), and alpha-lipoprotein levels in individuals with microalbuminuria to decrease, although glomerular filtration rate (GFR) levels in people with normo-albuminuria declined^39^. This finding is consistent with the results of studies on type 1 diabetes and the replacement of animal protein with plant protein^41,66,67^ The mechanism behind this effect may be related to the reduction of renal plasma flow^43^.

Inflammation may help explain renal dysfunction. According to the Multi-Ethnic Study of Atherosclerosis (MESA), inflammatory markers, including high-sensitivity C-reactive protein (hs-CRP) and E-selectin, were negatively correlated with a balanced diet pattern that includes fruits, vegetables, whole grains, and seafood. In contrast, the Western food pattern, which is rich in red meat and processed foods high in fat, increased inflammatory markers such as hs-CRP, interleukin-6 (IL-6), E-selectin, cytokine regulation of cellular adhesion molecule-1 (CAM-1), and vascular cell adhesion molecule-1 (VCAM-1)^68,69^.

The quantity of protein is similar in red, white, and processed meat from various sources, but the fatty acid composition differs. Compared to red and processed meat, white meat contains higher levels of monounsaturated fatty acids (MUFAs) and polyunsaturated fatty acids (PUFAs) and lower levels of saturated fatty acids (SFAs)^70^. Consumption of foods low in PUFAs is inversely related to inflammatory markers^71^ and can cause endothelial dysfunction^72,73^. The difference in their fatty acid composition is likely responsible for the superior benefits of white protein sources on renal function compared to red and processed meat. In summary, endothelial dysfunction can disrupt normal kidney function by affecting blood flow regulation, promoting inflammation and oxidative stress, and contributing to conditions like hypertension and diabetes, all of which can lead to renal dysfunction and kidney disease.

High levels of serum cholesterol are known to be a risk factor for diabetic nephropathy (DN) in type 2 diabetes (T2D) patients^74^. Reducing the intake of saturated fatty acids (SFAs) and increasing the intake of monounsaturated fatty acids (MUFAs) and polyunsaturated fatty acids (PUFAs) can lower total cholesterol (TC)^75^. In the present study, patients with higher scores for red and processed meat consumption had a higher intake of saturated fatty acids. However, this association was inverse for patients with higher scores for white meat consumption. Additionally, those with higher intakes of white meat sources had lower TC.

Moderate protein restriction is associated with decreased urinary albumin excretion rate (UAER) in type 2 diabetes^76,77^, although one study produced conflicting results^39^. It has been suggested that switching to white meat, such as chicken or fish, may be more advantageous than merely reducing overall protein intake^78^. An observational study found that consuming more protein sources from white meat, including chicken and fish, improves kidney function in diabetic patients, which aligns with the findings of a recent study^78^. The different amino acid composition of white meat and red meat may be one of the reasons for the advantages of substituting white meat for red meat^79^. Beef, for instance, has higher levels of arginine and glycine compared to chicken, which can affect kidney function^79^, although in patients with type 1 diabetes, plasma amino acid levels were not significantly different in those following a diet based on chicken and fish compared to beef^41^.

The present study has several strengths, including being the first to evaluate the association between types of meat and diabetic nephropathy (DN). Food intake was assessed using a valid and reliable food frequency questionnaire (FFQ), and sampling was conducted at a single center over an appropriate period. However, this study also had limitations that should be noted. It employed a case–control study design, which can introduce bias. All participants were female, limiting generalizability to males. The matching of the case and control groups was based on age and duration of diabetes, which may influence the results due to the presence of other confounding variables, although most confounding variables were considered in the analysis. Further investigations into the association between kidney function factors and the consumption of red, white, and processed meat could provide deeper insights.

To our knowledge, this is the first study to assess the association between the prevalence of DN and kidney function markers and the consumption of different types of meat. In conclusion, we observed that higher consumption of red and processed meat sources was linked to microalbuminuria, severe albuminuria, higher blood urea nitrogen (BUN) levels, and a higher odds ratio of DN. Given the inconclusive literature, we recommend further well-controlled RCT studies in different populations and ethnicities with other designs such as cohorts.

## Methods and materials

### Subjects

In this case–control study, a total of 210 participants, comprising 105 cases and 105 controls aged 30–65 years, with a 3- to 10-year history of type 2 diabetes (T2D), were referred to the Kowsar Diabetes Clinic in Semnan, Iran, between July and December 2016. The diagnosis of diabetes in this study was based on the American Diabetes Association's new diagnostic criteria, which include glycosylated hemoglobin (HbA1c) ≥ 6.5%, fasting blood glucose (FBG) ≥ 126 mg/dL, 2-h post-load blood glucose (2hrBG) ≥ 200 mg/dL, or, in a patient with classic symptoms of hyperglycemia or hyperglycemic crisis, a random plasma glucose ≥ 200 mg/dL (11.1 mmol/L)^80^. Participants with a previous history of cancer, autoimmune disorders, liver disease, coronary angiography, myocardial infarction, or stroke were excluded from the study. Additionally, individuals with poor responses to the Food Frequency Questionnaire (FFQ) or total energy intake below 500 or above 3500 kcal per day were excluded. All participants in this study read and signed written informed consent before participating. In 2016, the study protocol was approved by the ethics committee of Tehran University of Medical Sciences (TUMS) with the identification number IR.TUMS.REC.1395.2644.

### Anthropometric measurements

Body weight (kg) was measured while participants were unshod and wore light clothes, according to standard protocols. Body mass index (BMI) was calculated by dividing weight by the square of height (kg/m^2^).

### Dietary assessment

Dietitians assessed the dietary intake of participants over the past year through face-to-face interviews using a validated Food Frequency Questionnaire (FFQ)^81^. Dietary intakes were reported and recorded in terms of yearly, monthly, weekly, or daily frequency, and then converted to grams per day using household measurements. The obtained amounts were adjusted for energy intake using the residual method^82^. The calculation of macro- and micronutrient content and grams of foods was performed using NUTRITIONIST IV software (version 3.5.3.; N‐Squared Computing, Salem, OR, USA).

This study focused on the consumption of three different types of meat as extracted from the FFQ, measured in grams per day: (1) The red meat category included beef, lamb, and sheep/mutton, as well as organ meats like beef liver, kidney, tongue, and heart; (2) White meat comprised fish and poultry, including chicken and turkey; and (3) Processed meats included sausages, Kalbas, hamburgers, Mortadella, and canned fish.

### Assessment of DN

DN is defined as the ratio of urinary albumin to creatinine (ACR) ≥ 30 mg/g in a random spot urine sample^83^, and its’ diagnosis was confirmed by a clinician.

### Assessment of markers of kidney function

GFR is generally accepted as the best overall index of kidney function and is the most important parameter to determine in the clinical evaluation of kidney function. Normal GFR ≥ 90 mL/min/1.73 m^2^ and mild to severe GFR (< 90 mL/min/1.73 m^2^) is, by itself, sufficient for the diagnosis of chronic kidney disease (CKD), regardless of the presence or absence of other markers of kidney damage^84^.

Patients were then divided into two groups: ‘normal’, if BUN was ≤ 20, and ‘high’ if BUN was > 20^85^. Participants' albuminuria was determined based on ACR categories using the Epidemiology Collaboration (EPI) equation formula, as reported by Levey et al.^86^: Normal < 30 mg/g, micro 30–300 mg/g, and severe 300 mg/g.

### Assessment of blood pressure

Systolic and diastolic blood pressure (SBP, DBP) of participants were measured on the left arm, after 15 min of rest, by using an automatic sphygmomanometer.

### Blood parameters

Biochemical variables consisted of fasting blood sugar (FBS), HbA1c, 2hrBG, kidney function tests [BUN, total serum creatinine (Cr), albumin^6^], and lipid profile such as total cholesterol (TC), high-density lipoprotein cholesterol (HDL_C), low-density lipoprotein cholesterol (LDL_C), and triglycerides (TG), and were obtained from the most recent 3 months of participants medical records.

### Demographic variables and physical activity

Through a demographic questionnaire, demographic information including weight, height, age, medical history, and the type of drugs/medicines (including angiotensin-converting enzyme inhibitors (ACE), Beta-blockers, Metformin, Sulfonylurea, and Insulin) was obtained. The physical activity of the participants was assessed using the International Physical Activity Questionnaire (IPAQ). The scores of the IPAQ were categorized into three levels of physical activity: 'low physical activity' (point score < 600 metabolic equivalents (MET)/hour per week), 'moderate physical activity' (point score between 600 and 3000 MET/hour per week), and 'high physical activity' (point score > 3000 MET/hour per week).

### Statistical analysis

The normality of the quantitative variables was evaluated using the Kolmogorov–Smirnov test. Quantitative variables, including age, height, weight, and BMI, were compared between cases, and controls were described as mean ± standard deviation^9^, and for categorical variables, frequency (%) was used. Independent t-test and chi-square tests were used for quantitative variables and categorical variables across the low adherence and high adherence to the red, white, and processed meat between cases and controls, respectively. To characterize the distribution of the qualitative variables across the low adherence and high adherence to the red, white, and processed meat between cases and controls in an adjusted model, we used Analysis of Covariance (ANCOVA). Logistic regression was used to determine the association between food groups and odds of DN. Variables including age, energy intake, Albumin, hemoglobin, physical activity, history of cardiovascular diseases (CVD), and disease duration were controlled for in the adjusted model. SPSS software (Version 25, SPSS Inc., Chicago, IL, USA) was used to analyze data and P < 0.05 was considered statistically significant.

### Ethics approval and consent to participate

The study protocol was approved by the ethics committee of Tehran University of medical sciences (IR.TUMS.MEDICINE.REC.1401.811) and is acknowledged by authors. All participants signed a written informed consent.

### Statement

We state that all methods are based on the relevant guidelines and regulations.

## Data Availability

The data that support the findings of this study are available from Dr. Khadijeh Mirzaei but restrictions apply to the availability of these data, which were used under license for the current study, and so are not publicly available. Data are however available from the authors upon reasonable request and with permission of Dr. Khadijeh Mirzaei.

## Abbreviations

ARB: Angiotensin receptor blockers
ACIE: Angiotensin-converting enzyme inhibitors
ANCOVA: Analysis of covariance
AGEs: Advanced glycation end products
ACR: Albumin to creatinine
BUN: Blood urea nitrogen
BMI: Body mass index
Cr: Creatinine
CVD: Cardiovascular diseases
CKD: Chronic kidney disease
CAM-1: Cellular adhesion molecule
DN: Diabetic nephropathy
DM: Diabetes mellitus
DASH: Dietary approaches to stop hypertension
DBP: Diastolic blood pressure
ESRD: End-stage renal disease
EPI: Epidemiology collaboration
eGFR: Estimated glomerular filtration rate
FBS: Fasting blood sugar
FBG: Fasting blood glucose
FFQ: Food Frequency Questionnaire
GFR: Glomerular filtration rate
2hrBG: 2-H post-load blood glucose
HbA1c: Glycosylated hemoglobin A1c
HDL-C: High-density lipoprotein cholesterol
Hs-CRP: High-sensitivity C-reactive protein
LDL-C: Low-density lipoprotein cholesterol
IPAQ: International Physical Activity Questionnaire
IDDM: Insulin-dependent diabetes mellitus
IL-6: Interleukin 6
LPD: Low protein diet
MUFAs: Mono unsaturated fatty acids
MESA: Multi-Ethnic Study of Atherosclerosis
mTORC1: Mechanistic target of rapamycin complex 1
PRM: Processed red meat
PUFAs: Poly unsaturated fatty acids
ROS: Reactive oxygen species
SBP: Systolic blood pressure
SD: Standard deviation
SFAs: Saturated fatty acids
TNF-α: Tumor necrosis factor alpha
T2D: Type 2 diabetes
T2DM: Type 2 diabetes mellitus
TC: Total cholesterol
TG: Triglycerides
URM: Unprocessed red meat
UD: Usual diet
UAER: Urinary albumin excretion rate
VCAM-1: Vascular cell adhesion molecule 1
